# The tiger salamander as a promising alternative model organism to the axolotl for fracture healing and regenerative biology research

**DOI:** 10.1002/ar.70060

**Published:** 2025-10-08

**Authors:** Vivien Bothe, Nadia Fröbisch

**Affiliations:** ^1^ Museum für Naturkunde Berlin, Leibnitz Institute for Research on Evolution and Biodiversity Berlin Germany; ^2^ Department of Biology Humboldt University Berlin Berlin Germany

**Keywords:** Ambystoma, axolotl, metamorphosis, pathologies, regeneration, tiger salamander

## Abstract

Scientists have been captivated by the ability to regenerate, focusing on uncovering the mechanisms of epimorphic regeneration and applying them to human medicine. The axolotl (*Ambystoma mexicanum*) has become the most intensively studied model in tetrapod regeneration research, particularly concerning limb regeneration. This research has provided significant insights into signaling pathways and factors regulating limb regeneration. However, most regeneration studies focus on controlled, surgical amputation experiments under strict laboratory conditions. This practice has limited the available data on natural bite‐induced regeneration, which, however, is crucial for understanding the natural condition in wild populations and provides insights into the biology and evolution of regenerative capacities. Moreover, the axolotl's paedomorphic life history limits the generalization of findings to other salamander taxa. This study compares limb regeneration in axolotls and their metamorphosing sister taxon, the tiger salamander (*Ambystoma tigrinum*), across various ontogenetic stages to identify common and variable aspects of the regeneration process, providing a basis for future comparative studies in different salamander taxa. The results demonstrate that tiger salamanders have excellent regenerative capacity during the larval stage, which is in no way inferior to that of axolotls. Post‐metamorphic tiger salamanders are still able to regenerate limbs. However, there is a clear slowdown in the speed of regeneration and an increase in skeletal anomalies. Axolotls are often subject to bite attacks even in the adult stage. This leads to severe pathologies in the limb anatomy after regeneration, up to severely restricted movement or non‐functional limbs.

## 1 INTRODUCTION

For over a hundred years, the ability to rebuild lost body parts and organs in a seemingly perfect fashion has captivated scientists, and research efforts have focused on unlocking the underlying mechanisms of epimorphic regeneration and making them applicable to human medicine (Alvarado, 2000; Bely & Nyberg, 2010; Bölük et al., 2022; Nacu & Tanaka, 2011; Nye et al., 2003).

The axolotl limb is probably the most intensively studied body part with regard to tetrapod regeneration research (Gardiner et al., 2007; Kragl et al., 2009; Simon & Tanaka, 2013; Tanaka, 2003), though axolotls are also capable of tail, lens, heart, and liver regeneration (Echeverri & Tanaka, 2002; Ghosh et al., 1994; Mitashov, 1996; Oberpriller & Oberpriller, 1974; Reyer, 1954). The ease of axolotl husbandry in the laboratory has contributed to the fact that the axolotl became and still remains the vertebrate regeneration model organism par excellence. It was with the help of this model organism that it was possible to gain knowledge on relevant signaling pathways and factors regulating controlled growth and pattern formation during limb regeneration (Gerber et al., 2018; Glotzer et al., 2022; Haas & Whited, 2017; McCusker et al., 2015; Nacu et al., 2016; Torok et al., 1999).

When keeping this species in the laboratory, it quickly becomes apparent that it is not possible to house several individuals in a group together without risking slight or even severe injuries to the limbs and/or tail tips caused by conspecific biting. Aggressive behavior among larvae is well known for several *Ambystoma* species, including the axolotl, and conspecific biting continues in adult axolotls, regardless of sufficient habitat space and food supply (Bothe & Fröbisch, 2023; Bothe, Mahlow, & Frobisch, 2021; Semlitsch & Reichling, 1989; Thompson et al., 2014; Wildy et al., 2001).

Since Bryant et al. (2017) have shown that axolotl eventually lose their regenerative abilities following multiple successive amputations, using uncontrolled group housing can confound conclusions about regeneration. On the one hand, keeping axolotls separately once they develop limb buds and performing controlled, surgical amputation experiments on so‐called naive limbs is a rigorous approach to elucidate the mechanistic aspects of limb regeneration. On the other hand, group‐housed animals have the advantage of representing the more naturalistic scenario with intense inter‐ and intraspecific predation, including cannibalism, that many salamander species face in the wild (Bothe et al., 2025). However, very little is known about regeneration following natural bite injuries. In order to advance the field of regeneration research further, it is important to use both approaches, including transparent housing conditions, to tease apart evolutionary factors of the regeneration process, which might be subject to selection.

Another somewhat limiting factor in the regeneration research on axolotls is the highly derived paedomorphic life history pattern of this species (De Groef et al., 2018; Denoël et al., 2005; Voss & Shaffer, 1997). Never undergoing metamorphosis into an adult terrestrial animal under natural conditions, the axolotl retains a number of morphological and physiological larval characteristics throughout its entire lifespan. This life cycle is not widespread among salamanders and only occurs in some clades of the species‐rich urodele clade (amphiaweb.org), where metamorphosis and direct development are much more widespread (Duellman & Trueb, 1994; Inger et al., 1986; Petranka, 1998; Pyron & Wiens, 2011). While the axolotl has, without a doubt, been integral in establishing and propelling the field of regenerative biology forward, it remains largely unknown to what extent regenerative processes vary in salamander species with different life history patterns and habitats, and to what degree findings obtained from the axolotl can be generalized. It is therefore of great interest to look beyond the model organism and investigate both species closely related to the axolotl but exhibiting different life histories, as well as more distantly related salamander species in order to identify variations in regeneration processes. Ultimately, this will provide the basis for identifying the common program of tetrapod regeneration, evolutionary patterns, and aspects of regeneration that are subject to evolutionary change and selective pressure.

This study focuses on the comparison of the morphology and histology of limbs undergoing regeneration following conspecific attacks in the axolotl and its metamorphosing sister taxon, the cannibalistic tiger salamander (*Ambystoma tigrinum*). Comparisons are made at various ontogenetic stages spanning larval, metamorphic, and post‐metamorphic stages. We established a morphological and histological record of limb regeneration following conspecific biting, considering milder as well as severe injuries, allowing for an assessment of gross anatomical and tissue‐level organization of regeneration after natural bites. The results provide a framework for future investigations that can address definitive milestones and features of the regeneration process, such as the formation of an AEC, blastema formation, and resolving cell debris, the involvement of different regulating parameters such as nerves, the role of stem cells, the importance of positional information and cell identity, and of different tissue structures in comparative frameworks using salamander taxa with different ecologies and life histories.

## 2 MATERIALS AND METHODS

All procedures were conducted according to Directive 2010/63/EU, the guidelines of the Tierschutz‐Versuchstierverodnung (TierSchVersVO) and Tierschutzgesetz (TierSchG), and approved by the Landesamt für Gesundheit und Soziales (LaGeSo/ZH104). Animals were euthanized using Tricaine (MS222), and all efforts were made to minimize suffering.

### 2.1 Species and housing

#### 2.1.1 *Ambystoma mexicanum* (SHAW, 1798)

Adult Mexican axolotls are derived from the animal facility of the Museum für Naturkunde Berlin. Animals were housed in small groups of five to six individuals in big tanks (100 × 60 cm) at room temperature, maintaining a 12 h light to 12 h darkness cycle. They were fed every 2 to 3 days with axolotl‐specific pellets. Axolotls used in this study were between 4 and 5 years of age at the time of collection and ranged from ∼20 to 21 cm snout‐vent length.

#### 2.1.2 Ambystoma tigrinum (GREEN, 1825)

Tiger salamanders used for this study derive from a colony at the Museum für Naturkunde Berlin. After hibernation, mature animals (three males, five females) were kept together in an outdoor water basin in spring for mating and oviposition. Therein, housing was set up to closely resemble natural habitat conditions. Eggs were laid within a few days after the transfer of the animals to the outside facility. Approximately 14 days post‐oviposition, adult salamanders were removed from the basin shortly after the first larvae had hatched. After another 4 weeks, about 200 larvae were collected from the outdoor basin and transferred to indoor tanks at room temperature, maintaining a 12 h light to 12 h darkness cycle. In this approach, we enhanced control over conditions and daily inspection. Additionally, larvae were categorized by size into smaller transparent plastic containers with perforations, facilitating constant water flow within larger tanks. Initially, 10 to 15 larvae were housed together per box. As the study progressed, the number of larvae per container was reduced to ensure adequate space for the growing larvae. Water changes were conducted on a weekly basis, while larvae were fed every other day. Smaller larvae were fed with *Artemia nauplii*, whereas larger larvae were provided with a diet of both white and red mosquito larvae. At the onset of metamorphosis, additional land units were placed in the water, enabling the subadults to gradually transition to land movement. Towards the end of the metamorphosis, juvenile salamanders were transferred to a large tank equipped with both aquatic and terrestrial sections, receiving a diet of crickets three times per week.

### 2.2 Gross observations

#### 2.2.1 Ambystoma mexicanum

Adult axolotls kept in groups frequently caused bite wounds to body appendages of conspecifics. Consequently, limbs were either severed or injured, often repeatedly, even if the limb was already in a process of regeneration. Repeated injuries and subsequent healing endured for over a year. Animals were euthanized with 4% tricaine and fixed in fresh 4% formaldehyde (ROTI®Histofix 4%) for about 5 days.

#### 2.2.2 Ambystoma tigrinum

During the daily inspection of the tiger salamanders, particular attention was paid to injuries to the limbs caused by bites of conspecifics. Affected animals were separated into their own container to prevent further bite injuries, to allow for rest, and to ensure a record of identity. The regeneration process of the limb was documented at regular intervals using a ZEISS stereomicroscope SteREO Discovery.V20 and photographed using a ZEISS axiocam 506 color. If required for this procedure, salamanders were anesthetized with Tricain (MS 222, Sigma; 0.1% for larvae, 0.2% for juvenile postmetamorphs). In a previous breeding session, injured larvae were not separated from their conspecifics and raised in groups until the adult stage. There is no detailed documentation of the injury and healing history of these animals. For morphological examination, animals were euthanized with 2% tricaine and fixed in fresh 4% formaldehyde (ROTI®Histofix 4%) for about 48 h.

### 2.3 Clearing and staining

Salamander limbs were removed from the body, skinned, and double‐stained to visualize cartilaginous and bony skeletal elements. The protocol was modified according to (Ovchinnikov, 2009). The cartilaginous components were stained using a 0.015% Alcian blue solution for approximately 12 h at room temperature, followed by a thorough wash in an ethanol series. Maceration was performed in trypsin (0.1%, Sigma) for 2 to 5 weeks at 37°C. Bony skeletal elements were stained in 0.01% Alizarin red solution for approximately 4–5 h and washed afterward in a 30% glycerin solution. For long‐term storage, the samples were transferred to a 100% solution of glycerin.

### 2.4 Contrast‐enhanced micro‐CT imaging and analysis

Multiple limbs of adult tiger salamanders and axolotls were scanned in unstained condition to make ossified skeletal elements of the regenerated limbs visible. However, as x‐ray microtomography (μCT) scanning without prior tissue staining produced low inherent contrast of non‐mineralized soft tissues, suitable staining protocols using phosphotungstic acid (PTA) and Lugol's iodine (I2KI) were developed to produce images with better tissue‐specific gray contrasts. Selected limbs were stained in a 1% solution of iodine in distilled water for 7 days, followed by 1 week with 1.5% PTA in distilled water. The concentration of the staining solutions was gradually increased within the first days to protect the tissue while achieving the best possible staining results.

The limbs were examined through micro‐tomographic analysis at the Museum für Naturkunde Berlin by using a Phoenix nanotom X‐ray tube at 100 kV and 150 μA, generating 1440 projections with an exposure of 750 ms per picture. The effective voxel size was 18 μm. The cone‐beam reconstruction was performed using the datos|x‐reconstruction software (GE Sensing & Inspection Technologies GMBH Phoenix X‐ray datos|x 2) and the three‐dimensional reconstructions were visualized in VG Studio Max 3.5. (Volume Graphics Inc., Germany). Unstained bony structures were automatically segmented, and stained skeletal structures were segmented manually.

### 2.5 Histology

Limbs of adult axolotls were removed from the body and decalcified with 10% EDTA for approximately 4 weeks. Following embedding in paraffin, histological serial sections in a longitudinal orientation with a thickness of 6 μm were made using a microtome. Sections were stained with Heidenhain's Azan. To identify different types of tissue and blastema cells, sections were analyzed using transmitted light microscopy and photographed with a Leica DFC495 Digital Color Microscope Camera mounted on the Axioskop and the Leica Application Suite V 4.2. Software.

## 3 RESULTS

### 3.1 Cannibalistic behavior

**FIGURE 1:**
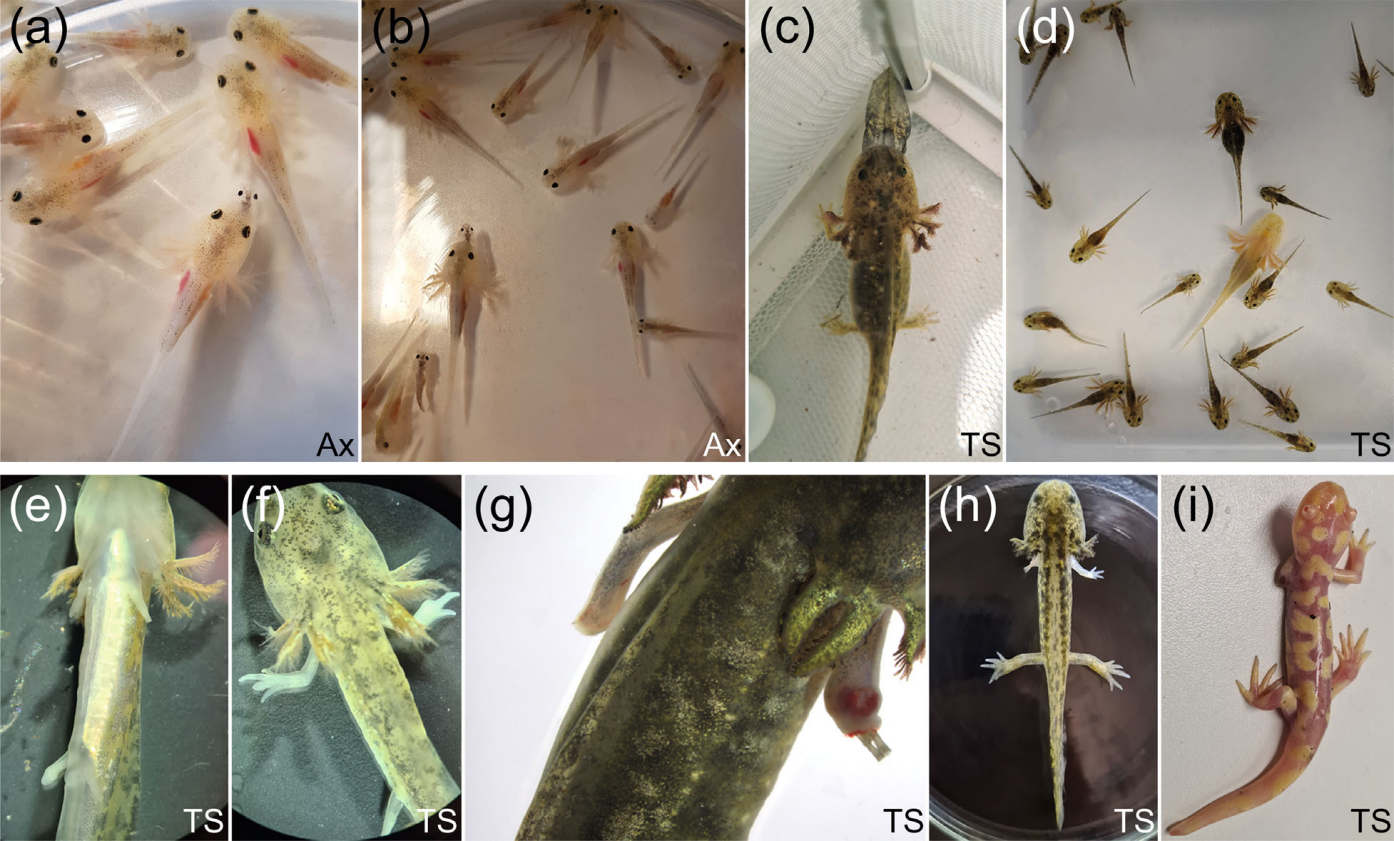
Cannibalistic behavior and bite injuries of *Ambystoma*. *A. mexicanum*: (a, b) Smaller individuals eaten by conspecifics. *A. tigrinum*: (c) Smaller individual eaten by a conspecific. (d) Size differences between non‐cannibalistic and cannibalistic morphs. (e–i) Individuals with simultaneous bite injuries to several limbs. Ax = Axolotl (*A. mexicanum*), TS = Tiger salamander (*A. tigrinum*).

**FIGURE 2:**
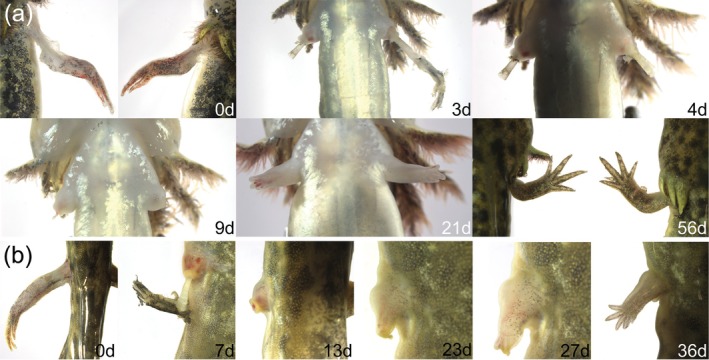
Morphological progress of limb regeneration of *A. tigrinum* larvae after limb loss. (a) Right forelimb. (b) Left forelimb. (c) Right hind limb. Larvae are about 8 months old. 0 h represents the day of collection and separation.

**FIGURE 3:**
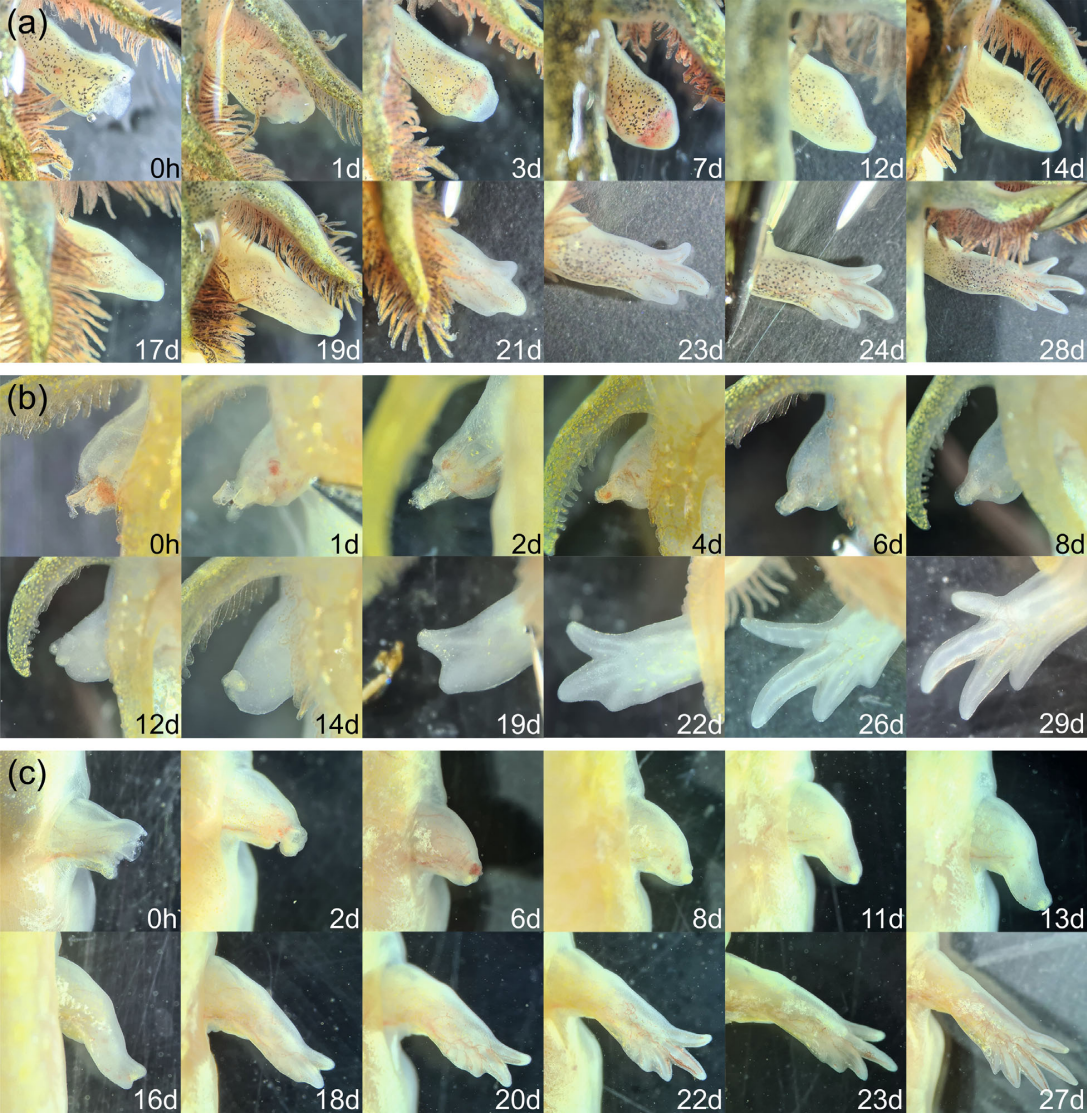
Regeneration progress of *A. tigrinum* larvae after severe contusions caused by conspecifics. (a) Both forelimbs are seriously injured. Larva is about 5 months old. (b) Seriously injured left hind limb. Larva is about 7 months old. 0 h represents the day of collection and separation.

**FIGURE 4:**
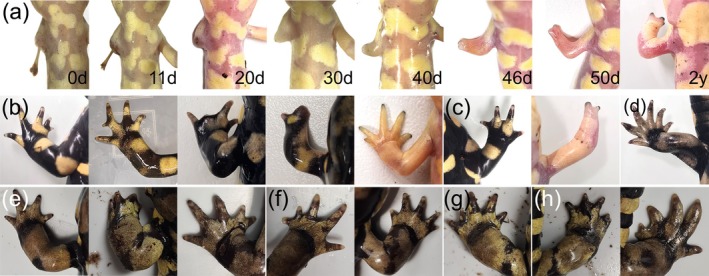
Regeneration of postmetamorphic *A. tigrinum*. (a) Regeneration progress of a subadult resulting in limb pathology, left forelimb. Subadult is about 20 months old. (b–h) Regenerated limbs after bite injuries with various malformations. (b, c) Forelimbs of subadults. (d) Hind limb of a subadult. (e, f) Forelimbs of adults. (g, h) Hind limbs of adults. 0 h represents the day of collection and separation.

Aggressive behavior towards conspecifics is pronounced in *A. mexicanum* and the closely related species *A. tigrinum*. Despite a sufficient food supply and a spacious housing situation, bite incidents occurred on a regular basis. Aggressive behavior was particularly pronounced in larvae, but also present in subadult and adult animals. Some attacks led to comparatively minor tissue damage, such as skin abrasions, contusions (Figure 8a,b), or more severe injuries (Figure 3a,b). In other incidents, fingers and toes or larger parts of limbs were severed. Therein, amputations took place in the autopod, zeugopod, or stylpod area, close to the body (Figures 1g–i and 2, 4, 8c,d). Smaller individuals or those already restricted in movement due to prior injuries were frequently targeted in repeated biting attacks. This frequently resulted in multiple injuries and subsequent regeneration processes affecting several, or in a few instances, all four limbs simultaneously (Figure 1e–i).

Cannibalistic behavior resulting in the complete consumption of conspecifics is also common in both species (Figure 1a–d), but much more frequent in *A. tigrinum*. In this species, this behavior leads to the development of two distinct morphs (Collins & Cheek, 1983), a phenomenon that has been reported not only under laboratory conditions but in the wild as well (Lannoo & Bachmann, 1984). The normal, non‐cannibalistic morph and a cannibalistic morph are characterized by a significantly larger body size, wider head shape, and hypertrophic dentition (Pedersen, 1991; Reilly et al., 1992; Figure 1d).

### 3.2 Regeneration abilities

#### 3.2.1 Tiger salamander

##### Regeneration process and externally visible malformations in larvae

The regenerative abilities of young tiger salamander larvae are remarkably good. The regeneration process closely mirrors that described for axolotls. The initiation of the wound healing process occurs within the first hours post‐injury. A wound epidermis forms on the amputated stump, providing protection to the injured tissue against external influences, followed by the development of an apical epithelial cap (AEC), controlled apoptosis, and the formation of a blastema—a cluster of undifferentiated cells beneath the AEC. An early regenerating limb bud becomes visible after approximately 1 week, followed by a palette stage, and the first differentiation can be monitored after approximately 2 weeks. Subsequent growth leads to the successive emergence of fingers or toes. It takes about 4 weeks until a new limb that has the normal structure and shape is fully developed (Figures 2 and 3). At this point, the regenerated limb still remains significantly smaller and subsequently undergoes rapid ontogenetic allometric growth until, after another 4 weeks, it reaches the proportionally appropriate size of the unamputated limb. At this point, the regenerate grows isometrically with the rest of the animal (Figure 3a).

Particularly noteworthy are the regenerative capacities in severely injured limbs that were not entirely removed from the body during the bite attack (Figure 2). Those parts of the limbs that were subject to severe damage from bruises and bites, including muscles and nerves, do not undergo recovery by tissue healing, despite still being attached to the limb. Instead, within a few days, the affected body part is shed, leaving only the healthy part of the limb. The regeneration process is then initiated on the remaining stump, following the usual steps of limb regeneration.

Limbs that are regenerated during the larval stage are usually indistinguishable from unregenerated limbs, even after severe injuries and rarely exhibit noticeable anatomical abnormalities. Occasionally, instances of supernumerary or missing digits occur (Figure 2a/56d).

##### Regeneration process and externally visible malformations in subadults and adults

The regenerative capabilities of tiger salamanders are clear but diminish after metamorphosis. The regrowth of missing limbs is significantly slower compared to the larval stage. In the case example documented here, even after more than 7 weeks, the final appearance of the limb does not seem to have been attained, and the regeneration process is still ongoing (Figure 4a). It should also be noted that protruding bones that remain after the injury are shed after a few days, similarly to what is observed in the larvae, leaving behind a fairly smooth limb stump on which the actual regeneration process is initiated. Although lost limbs grow back, they are much more prone to displaying externally visible skeletal anatomical defects, particularly in the form of extra or missing fingers and toes (Figure 4).

It is important to highlight that the limb pathologies observed in the adult animals depicted here can only tentatively be ascribed to past regeneration processes, as there is no further documentation regarding bite injuries, severity of injury, and age at the time of injury. However, it is noteworthy that most anatomical abnormalities rarely seem to significantly restrict the movability of the limbs or effectiveness of the locomotion of the animals.

##### Internal examination of limb skeletal anatomy following regeneration

**FIGURE 5:**
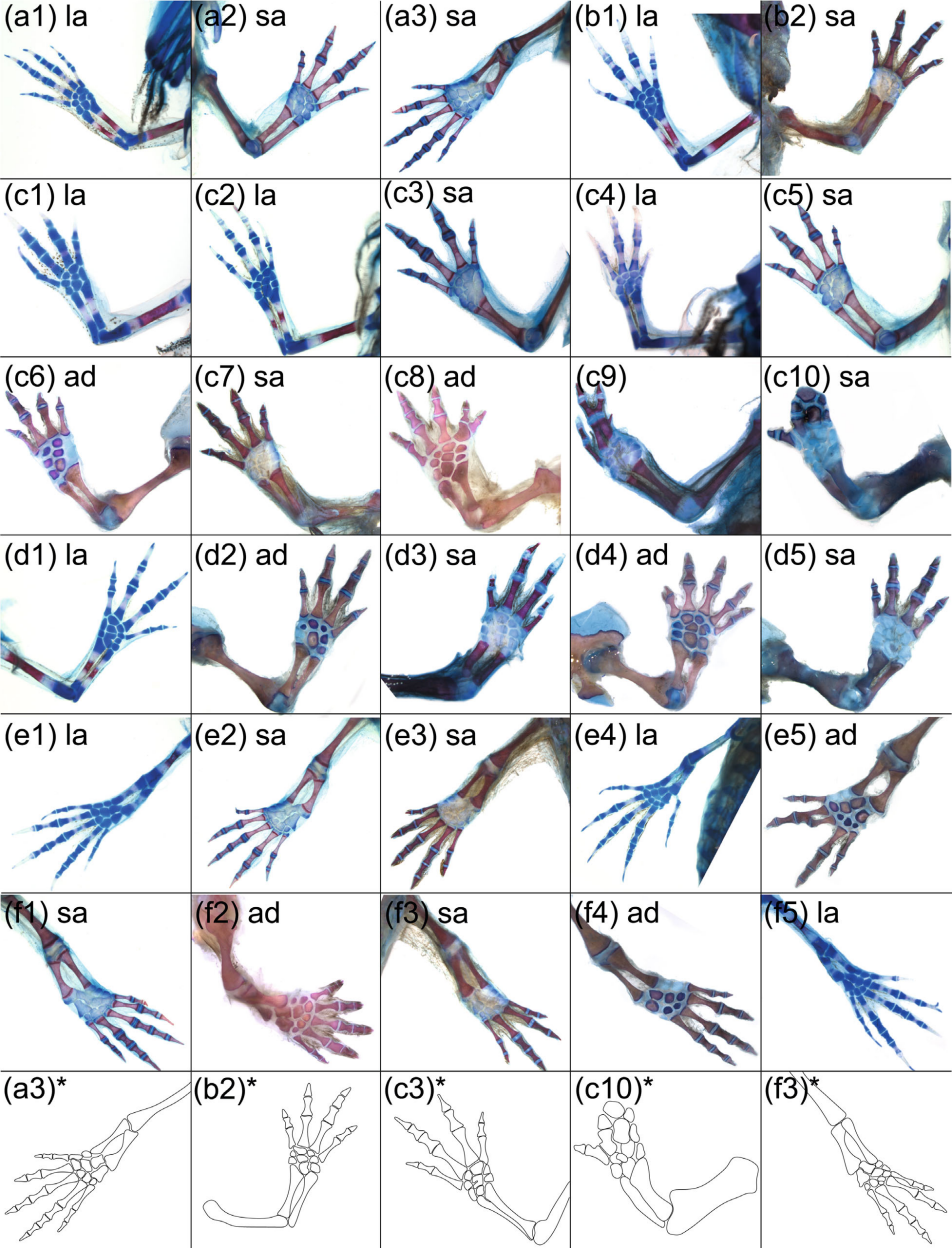
Cleared and stained limbs depicting aberrant skeletal anatomy in *A. tigrinum*. Ossified skeletal elements appear red, cartilaginous elements blue. (a) Normal not regenerated limbs. (b1) Normal skeletal anatomy following regeneration. (b2) Irregularly shaped humerus following regeneration. (c–f) Limbs with skeletal anomalies following regeneration. (c1–d5) Left and right forelimbs. (c1) Deviating number of carpal bones: 7 carpals. (c2) Deviating number of carpal bones: 9 carpals. (c3) Constricted carpal bone (centrale). (c4) Deviating phalangeal formula: 0‐2‐2‐2. (c5) Deviating phalangeal formula: 2‐2‐2‐2. (c6) Deviating phalangeal formula: 1‐1‐2‐1. (c7) Extra digit with two elements between digits III and IV. (c8) Bifurcated digit formed through two phalangeal elements adjacent to metacarpal 1. (c9) Malformed autopod including misshaped carpal bones (basale commune and distal carpalia d3), missing digit (probably digit 1), fused digits II and III, misshaped metacarpal II, and deviating phalange formula: x‐2‐2‐2. (c10) Bulky humerus, bulky radius, and ulna. Malformed autopod with deviating number of carpal bones: 7 carpals, and deviating phalangeal formula: 1‐1‐1‐1 with joined phalanges I and III. (d1) Deviating number of carpal bones: 9 carpals and noticeably constricted intermedium. (d2) Deviating number of carpal bones: 7 carpals and deviating phalangeal formula: 2‐2‐3‐1. (d3) Deviating phalangeal formula: 2‐3‐3‐3. (d4) Supernumerary digit IV with three elements. Missing phalangeal element on digit III. Bifurcated digit I. Noticeably constricted distal carpal d4. (d5) Bulky humerus, bulky radius, and ulna. (e1–f5) Left and right hind limbs. (e1) Deviating number of tarsal bones: 8 tarsals. (e2) Deviating phalangeal formula: 2‐3‐3‐3‐2. (e3) Deviating phalangeal formula: 2‐2‐3‐3‐2. (e4) Missing toe V. (e5) Distal fusion of metatarsal II and III whereby only four instead of five toes are formed. Missing phalangeal element on digit IV. (f1) Deviating phalangeal formula: 2‐2‐3‐3‐3. (f2) Deviating phalangeal formula: 2‐2‐3‐3‐2. (f3) Missing digit I. Missing phalangeal element on digit III and IV. Deviating number of tarsal bones: 10 tarsals. (f4) Missing digit, reduced distal tarsal d5, and missing phalangeal element on digit IV. (f5) Deviating phalangeal formula: 1‐2‐3‐4‐3. Bifurcated distal phalangeal element on digit II. A3*, B2*, C3*, C10*, D3* and F3*) Schematic illustrations. la = larval stage, sa = subadult stage, ad = adult stage.

Internal examination of limb anatomy confirmed that abnormalities in skeletal structure after completed regeneration occur more frequently in postmetamorphic tiger salamanders than in larvae, but they also revealed deviations in regenerated limb anatomy in larvae that are not externally apparent (Figure 5).

The standard phalangeal formula of *A. tigrinum* is 2‐2‐3‐2 with eight carpals in forelimbs (Figure 5a1, a2) and 2‐2‐3‐4‐2 with nine tarsals in hind limbs (Figure 5a3). In the larval stage, numerous limbs display near‐perfect regeneration following injury, irrespective of the severity of the injury (e.g., following complete amputation through a bite in the zeugopod area, Figure 5b1). Even in cases of severe injuries resulting in the shedding of necrotic tissue (Figure 3a), flawless regeneration of the missing parts of the limb can occur (Figure 5b2). In the example presented here, only the stylopod of the right forelimb exhibits a slightly bulky, irregular shape of the humerus instead of the typical narrowing in the diaphysis seen in non‐regenerated long bones. Moreover, bulkier long bones are more prevalent in regenerated limbs when amputation occurred in the stylopod or zeugopod after metamorphosis (Figure 5c10, d5) rather than in the larval stage.

Anomalies in the autopod are more variable owing to the increased number of skeletal elements. Frequently occurring deviations from the normal anatomy are missing carpals (Figure 5c1, c5, d2) and tarsals (Figure 5e1), supernumerary carpals (Figure 5c2, d1), noticeably constricted carpals (centrale) (Figure 5c3), missing digits (Figure 5e4) and missing phalanges in forelimbs (Figure 5c4, c5, c6, d2, d3) and hind limbs (Figure 5e2, e3, f2,f5), extra digits (Figure 5c7, d4), and missing toes (Figure 5f3, f4). In *A. tigrinum*, carpal ossification typically begins only after metamorphosis, during the late juvenile to early adult stages, usually around the time of sexual maturation (2–3 years of age). The carpal bones ossify in a sequential manner, with the radiale and tibiale generally among the last elements to ossify (Jia et al., 2022). This is the reason why the preaxial carpal bones in particular are not yet (fully) ossified in some subadult and adult animals shown here. Moreover, the incomplete ossification could also be related to incomplete regeneration and remodeling of the skeletal elements in the regenerated limbs.

Furthermore, more severe anatomical malformations occasionally occur, such as bifurcated digits (Figure 5c8, d4), distal fusion of metatarsals (Figure 5e5), or bifurcated distal phalangeal elements (Figure 5f5). In the most severe cases, regeneration results in pathological anatomical structures of the distal part of the limb (Figure 5c9, c10). For detailed information on the particular malformation, see the figure description of Figure 5.

**FIGURE 6:**
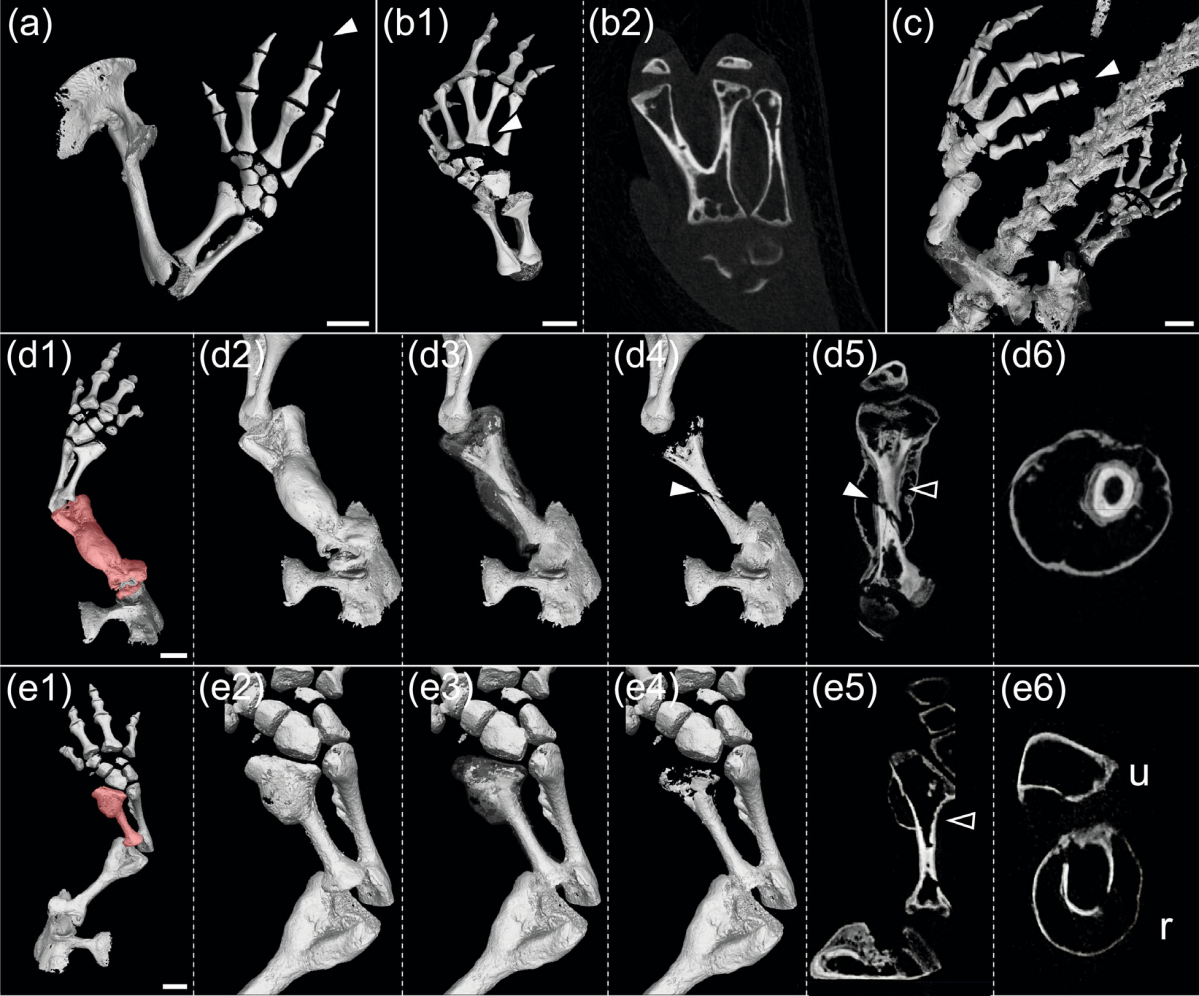
Internal limb skeletal anatomy of limbs of adult *A. tigrinum* after regeneration (a–c) and of forelimbs after fracture healing (d, e). Micro CT scans in an unstained condition and 3D reconstructions. (a) Deviating forelimb phalangeal formula: 3‐3‐3‐3. (b) Bifurcation of metatarsal IV resulting in six toes. (c1) Incomplete ossification of toe II. (d) Bulky humerus in the left forelimb. Bony callus formed around broken long bone. (e) Bulky radius in the right forelimb. Bony callus formed around injured distal end of the long bone. Filled arrows point to the anomalies or fracture sites. r = radius, u = ulna. Unfilled arrows display cross‐sectional planes. Scale bars represent 2 mm.

**FIGURE 7:**
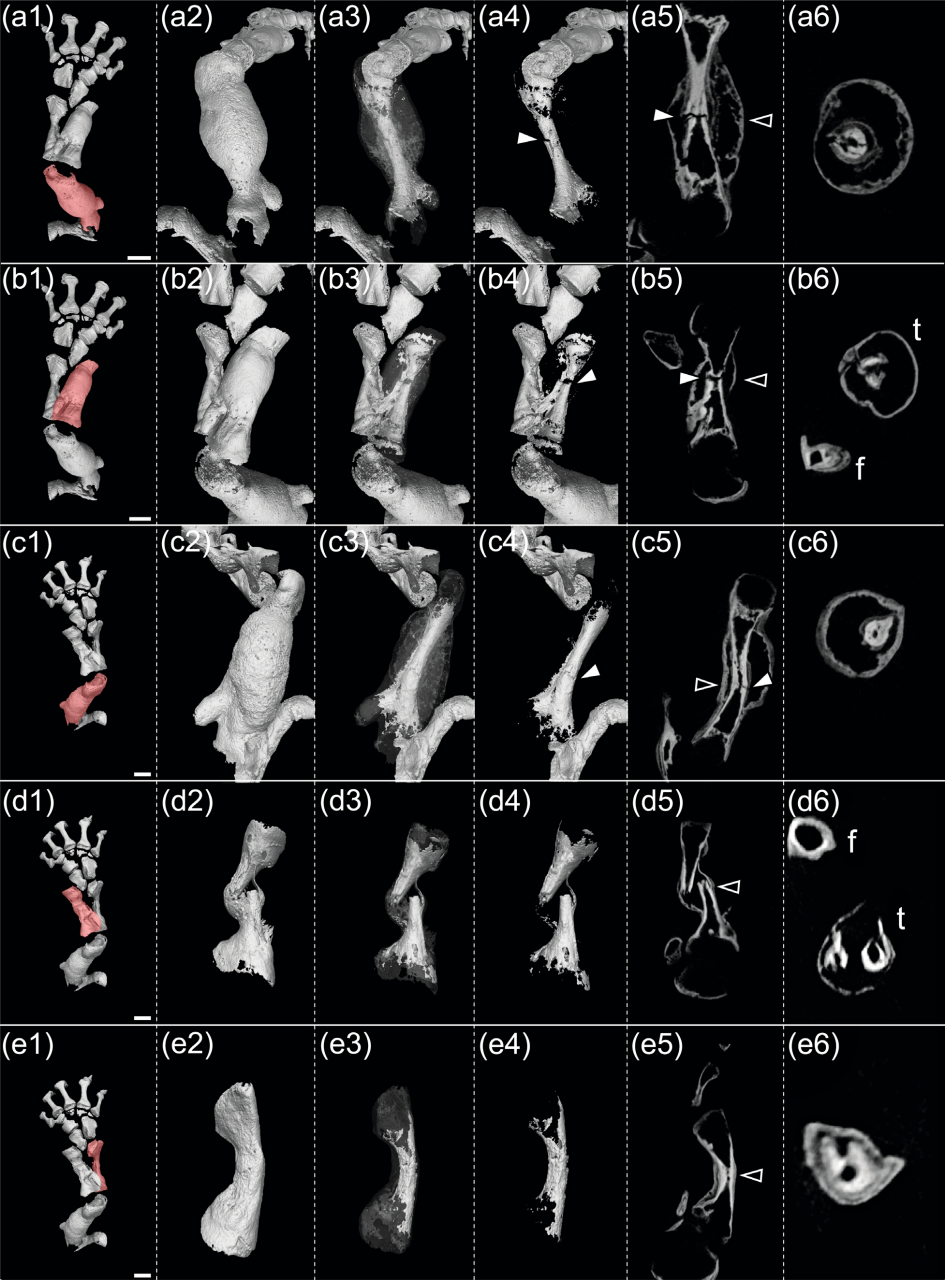
Internal limb skeletal anatomy of hind limbs of adult *A. tigrinum* after fracture healing. Micro CT scans in an unstained condition and 3D reconstructions. (a) Bulky femur in left hind limb. Bony callus formed around broken long bone. (b) Bulky tibia in left hind limb. Bony callus formed around broken long bone. (c) Bulky femur in right hind limb. Bony callus formed around broken long bone. (d) Bulky tibia in right hind limb. Bony callus formed around broken long bone. Ends of fractured elements are severely misaligned. Filled arrows point to the fracture sites. t = tibia, f = fibula. Unfilled arrows display cross‐sectional planes. Scale bars represent 2 mm.

The micro CT scan of two adult tiger salamanders, which had lived their entire life in group housing with conspecifics, revealed minor skeletal anomalies. A phalangeal phalanx is missing on digit III (Figure 6a), a bifurcation of metatarsal IV results in six toes (Figure 6b), and one phalangeal element on digit II exhibits incomplete ossification (Figure 6c). One individual shows extensive fracture healing in all four extremities (Figures 6d,e and 7), although no external abnormalities of the limbs were apparent. In the right forelimb, the distal end of the radius is surrounded by a bony callus. In the humerus of the left forelimb (Figure 6d, white arrow), as well as in the femurs and tibias of both hind limbs, fractures of the long bones could be identified (Figure 7a–d, white arrows). These fracture sites are surrounded by a massive bony callus. In one case, the fracture ends are severely misaligned, and the callus does not completely encase the fracture site (Figure 7d).

#### 3.2.2 Adult axolotl

##### Regeneration process and externally visible malformations in adults

**FIGURE 8:**
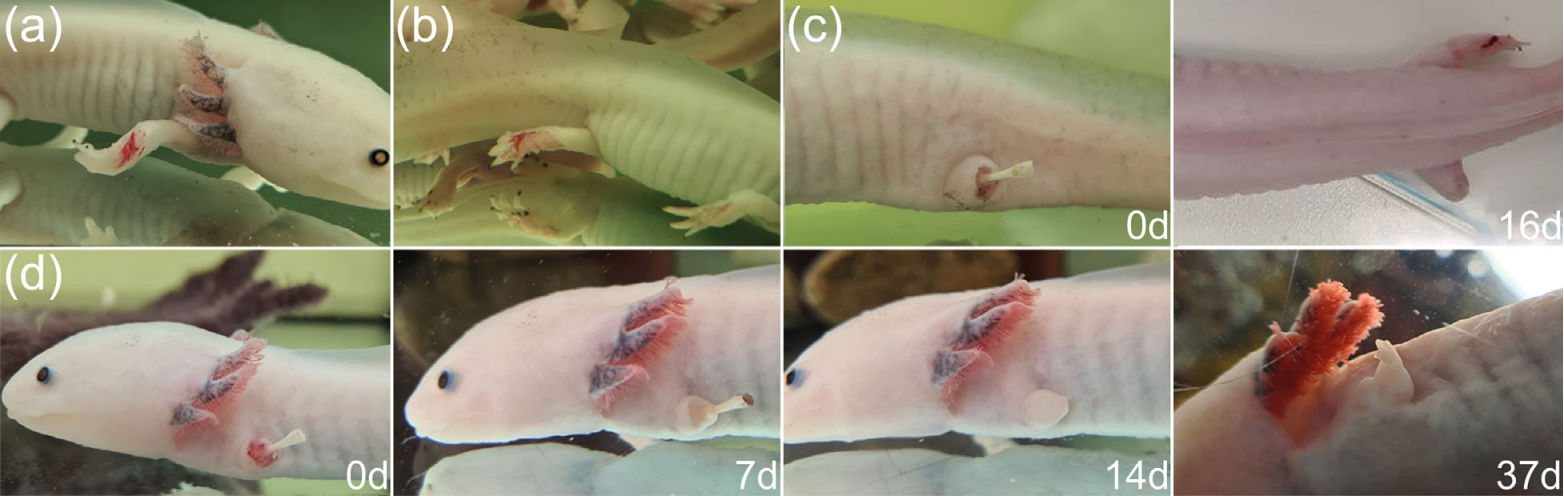
Limb regeneration after severe biting attacks by conspecifics. (a, b) Skin injuries and bruising of limbs. (c, d) Severed limb with protruding femur/humerus. Associated regeneration progress after 16/37 days. 0 h represents the day of isolation from conspecifics.

The outstanding regenerative capabilities following limb amputation of axolotl larvae are widely acknowledged and thoroughly documented and are not the focus of this investigation. Instead, the focus was directed towards limb pathologies arising from severe injuries inflicted by conspecifics (Figure 8a,b).

In a manner comparable to tiger salamanders, adult axolotl limbs that sustain severe injury rather than complete detachment following a bite attack are shed. This process unfolds progressively. Initially, necrotic soft tissue is shed, followed by the remaining exposed bone (Figure 8c,d). Eventually, only a smooth limb stump remains, adorned with healthy tissue from which the regeneration process commences. First, a smaller limb, referred to as a “tiny limb” (Wells et al., 2021), forms (Figure 8/37d), eventually maturing into a limb of its original size.

**FIGURE 9:**
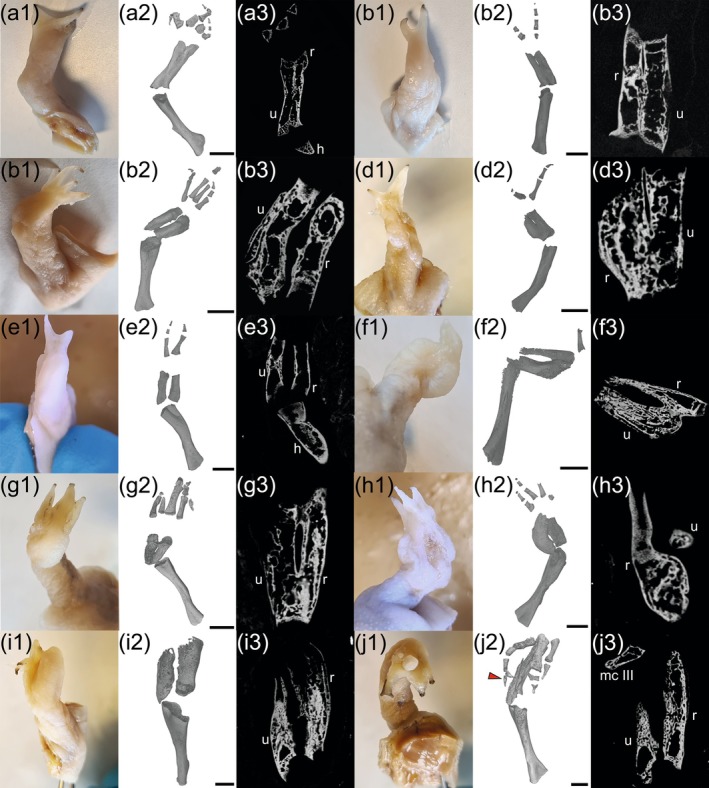
Internal limb skeletal anatomy after regeneration of adult *A. mexicanum*. Micro CT scans and 3D reconstructions of left (a, c, e, g, and i) and right forelimbs (b, d, f, h, and j) in an unstained condition. (a1–j1) External limb morphology. (a2–j2) Associated 3D models of unstained CT scans. (a3–j3) Associated longitudinal sections of selected areas. (a) Complete fusion of radius and ulna. Incorrect digit anatomy including missing digits and phalanges. (b) Fusion of radius and ulna in the proximal region. Incorrect digit anatomy including missing digits and phalanges. (c) Bulky long bones in the zeugopod area. Missing phalangeal elements. (d) Misshapen and fused long bones in the zeugopod. Incorrect digit anatomy including missing digits and phalangeal elements. (e) Bulky radius and ulna. Incorrect digit anatomy including missing digits and phalangeal elements. (f) Bulky radius. Distal fusion of radius and ulna. Digits are almost completely absent except for one phalangeal element. (g) Distal and proximal fusion of bulky‐shaped radius and ulna. Incorrect digit anatomy including missing phalangeal elements. (h) Severely malformed radius and bulky ulna. Incorrect digit anatomy including missing phalangeal elements. (i) Bulky radius and ulna. Complete absence of ossified autopod elements. (j) Incorrect digit anatomy including missing digit and distal bifurcation of digit (red arrow). h = humerus, mc = metacarpal, r = radius, u = ulna. Scale bars represent 5 mm.

**FIGURE 10:**
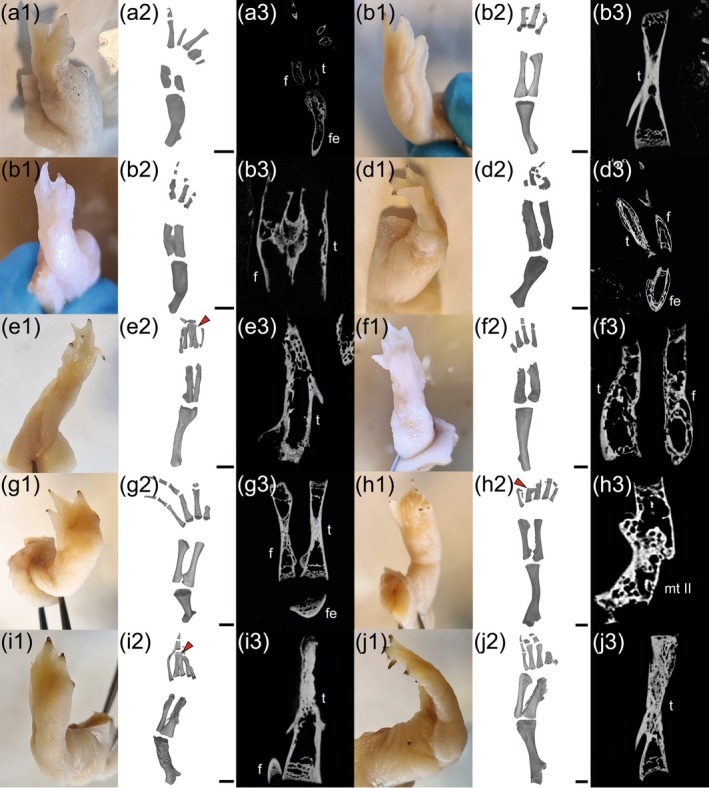
Internal limb skeletal anatomy after regeneration of adult *A. mexicanum*. Micro CT scans and 3D reconstructions of left (a, c, e, g, and i) and right hind limbs (b, d, f, h, and j) in an unstained condition. (a1–j1) External limb morphology. (a2–j2) Associated 3D models of unstained CT scans. (a3–j3) Associated longitudinal sections of selected areas. (a) Femur thickened in the distal area. Shortened and thickened long bones in the zeugopod. Significantly shortened digits caused by missing phalangeal elements. (b) Stylopod and zeugopod appear unregenerated. Incorrect digit anatomy including missing digits and phalangeal elements. (c) Femur thickened in the distal area. Thickened long bones in the zeugopod. Central fusion of tibia and fibula. Incorrect digit anatomy including missing digits and phalangeal elements. (d) Slightly thickened and irregularly shaped tibia and fibula. Incorrect digit anatomy including missing digits and phalangeal elements. (e) Distal fusion of metatarsal II and III (red arrow). Missing phalangeal elements in all digits. (f) Slightly thickened tibia and fibula. Incorrect digit anatomy including missing digits and phalangeal elements. (g) Stylopod, zeugopod, and digit I and II appear unregenerated. Incorrect digit anatomy including missing digits and phalangeal elements. Incorrect digit anatomy of digit III and IV caused by missing phalangeal elements. Digit 5 is completely absent. (h) Stylopod and zeugopod appear unregenerated. All digits shortened caused by missing phalangeal elements. Deformed metatarsal element (red arrow). (i) Thickened humerus. Irregular distal ends of tibia and fibula. Fusion of metacarpals III and IV (red arrow). Missing phalangeal elements in all digits. (j) Femur, fibula, and digit V appear unregenerated. Missing phalangeal elements in digits I–IV. f = fibula, fe = femur, mt = metatarsal, t = tibia. Scale bars represent 5 mm.

**FIGURE 11:**
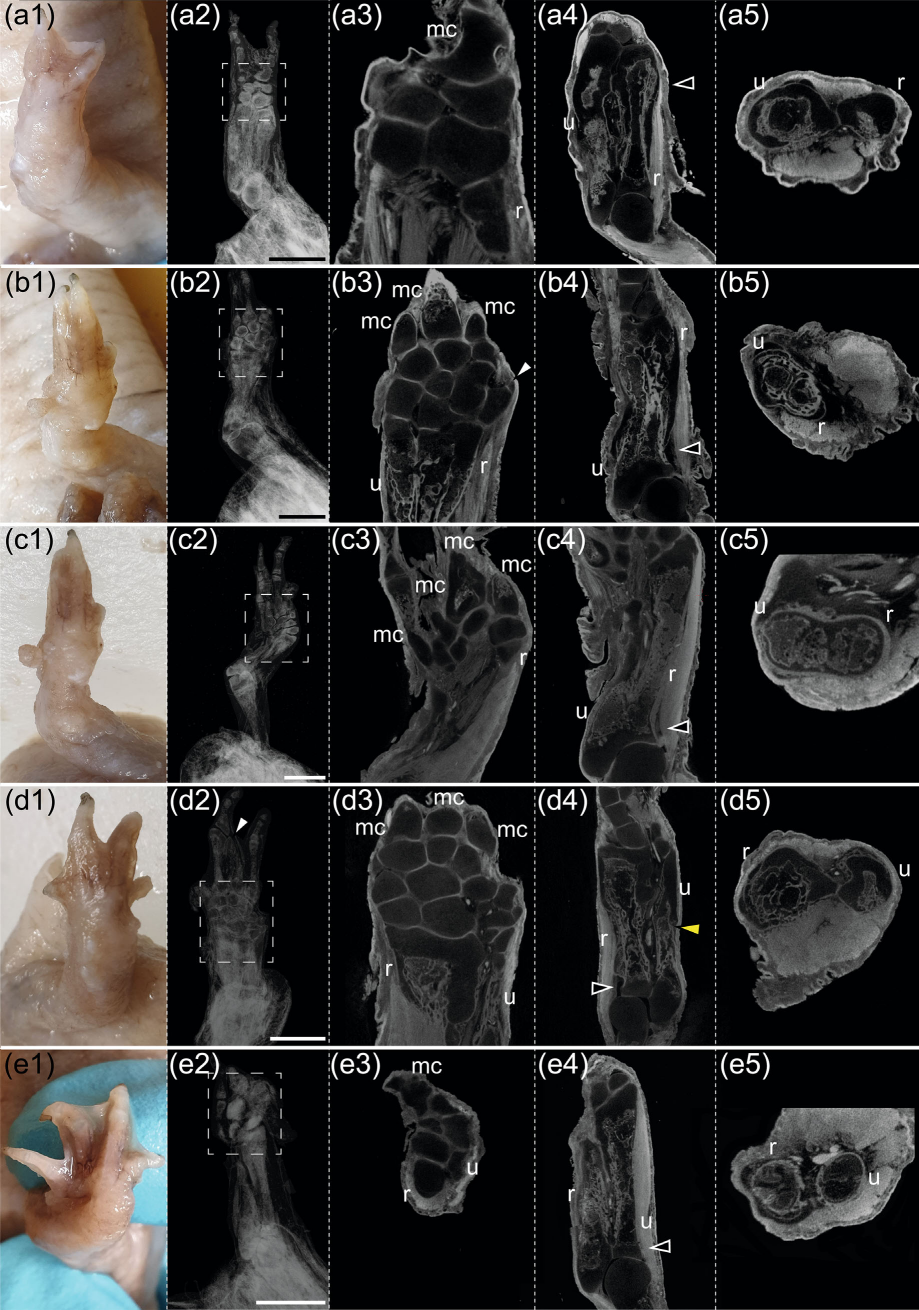
Internal limb skeletal anatomy after regeneration of adult *A. mexicanum*. Micro CT scans and 3D reconstructions of left (a–c) and right forelimbs (d, e) stained with iodine and PTA. (a1–e1) External morphology. (a2–e2) Semi‐transparent view. (a3–e3) Longitudinal sections of the carpal bone area. (a4–e4) Longitudinal sections of the zeugopod area. (a5–e5) Transversal sections of the zeugopod area. (a) Humerus thickened in the distal area. Bulky and misshaped radius and ulna. Deviating number of carpal bones: 4 carpals. Only two digits. (b) Fusion of long bones in the zeugopod. Abnormal alignment of the carpal bones. Only fingers III and IV developed. Finger II is only formed by incomplete metacarpal. The base of finger I can only be recognized by a vestigial metacarpal I (white arrow). (c) Proximal fusion of radius and ulna. Abnormal alignment and malformation of the carpal bones in the post‐axial area. Malformed digit I. (d) Distal thickening of the radius. Fusion of metacarpal II and III (white arrow). Several small supernumerary carpal bones in the post‐axial area. (e) Deviating number of carpal bones: 4 carpals. Dashed squares mark zoom‐in regions. Unfilled arrows display cross‐sectional planes. h = humerus, mc = metacarpal, r = radius, u = ulna. Scale bars represent 5 mm.

**FIGURE 12:**
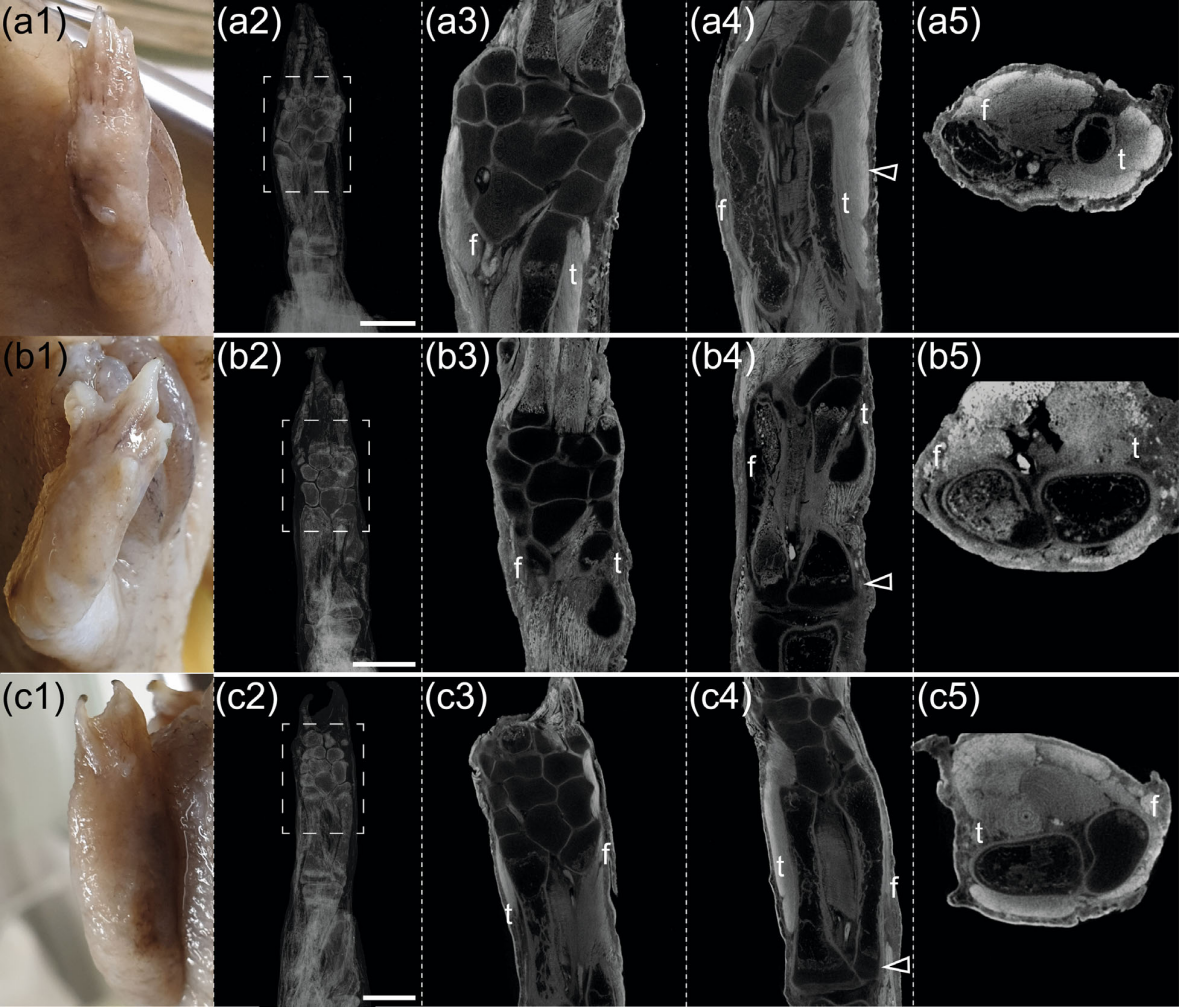
Internal limb skeletal anatomy after regeneration of adult *A. mexicanum*. Micro CT scans and 3D reconstructions of left (a and b) and right hind limbs (c) stained with iodine and PTA. (a1–c1) External morphology. (a2–c2) Semi‐transparent view. (a3–c3) Longitudinal sections of the carpal bone area. (a4–c4) Longitudinal sections of the zeugopod area. (a5–c5) Transversal sections of the zeugopod area. (a) Normal appearance of tarsal bone anatomy. Digit I consists only of a vestigial metacarpal I (white arrow). (b) Deviating number of tarsal bones: 8 tarsals. Missing digit. Digits are shortened by missing phalangeal elements. (c) Stylopod and zeugopod appear unregenerated. Deviating number of tarsal bones: 8 tarsals. Incorrect digit anatomy including missing digits and phalangeal elements. Dashed squares mark zoom‐in regions. Unfilled arrows display cross‐sectional planes. f = fibula, fe = femur, t = tibia. Scale bars represent 5 mm.

**FIGURE 13:**
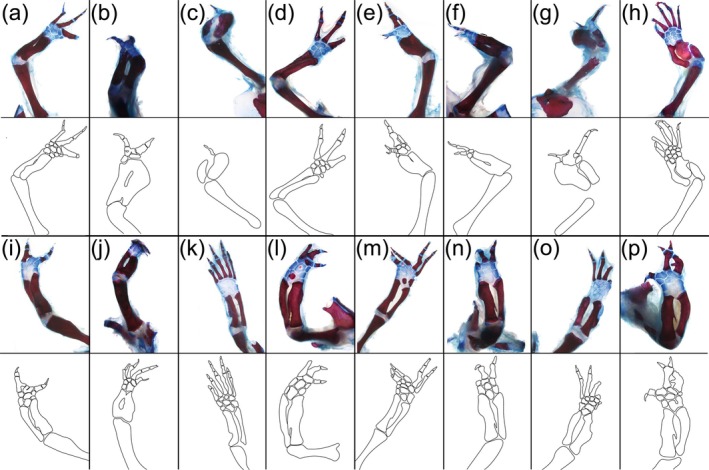
Internal limb skeletal anatomy after regeneration of adult *A. mexicanum*. Clearing and staining with associated schematic drawings. (a–d) Left forelimbs. (e–h) Right forelimbs. (i–l) Left hind limbs. (m–p) Right hind limbs. (a) Proximal fusion of radius and ulna. Deviating number of carpal bones: 9 carpals. Incorrect digit anatomy including missing digits and phalangeal elements. (b) Proximal and distal fusion of shortened and bulky radius and ulna. Deviating number of carpal bones: 2 carpals. Incorrect digit anatomy including missing digits and phalangeal elements. (c) Extremely shortened and shaped bulky‐shaped radius and ulna. Severely pronounced deformity of autopod area. Complete absence of carpal bones. One rudimentary digit. (d) Deviating number of carpal bones: 7 carpals. Incorrect digit anatomy including missing digits and phalangeal elements. (e) Complete fusion of zeugopod elements. Abnormal shape, alignment, and deviating number of carpal bones: 7 carpals. Incorrect digit anatomy including missing digits and phalangeal elements. Distal bifurcation of digit I. Distal fusion of metacarpals II and III. (f) Distal and proximal fusion of radius and ulna. Abnormal shape, alignment, and deviating number of carpal bones: 2 carpals. Incorrect digit anatomy including missing digits and phalangeal elements. (g) Misshaped humerus. Severe deformation of radius and ulna. Severely pronounced deformity of autopod area with one carpal bone and incorrect digit anatomy including missing digits and phalangeal elements. (h) Additional post‐axial element in the zeugopod. Deviating number of carpal bones: 10 carpals. Incorrect digit anatomy including extra digit, missing phalangeal elements, and distal fusion of digit II and IV. (i) Distal thickening of the humerus. Deviating number of tarsal bones: 7 tarsals. Incorrect digit anatomy including missing digits, missing phalangeal elements, and proximal branching of a metacarpal. (j) Thickened femur. Proximal fusion of shortened tibia and fibula. Tibia thickened. Deviating number of tarsal bones: 3 tarsals. Incorrect digit anatomy including missing digits and phalangeal elements. (k) Deviating number of tarsal bones: 8 tarsals. Incorrect digit anatomy including missing digits and missing phalangeal elements. (l) Distal thickening of the femur. Deviating number of tarsal bones: 8 tarsals. Abnormal ossification of three tarsal bones. Incorrect digit anatomy including missing digits and phalangeal elements. (m) Deviating number of tarsal bones: 10 tarsals. Abnormal ossification of three tarsal bones. Incorrect digit anatomy including missing digits, phalangeal elements, and deformed metacarpals. (n) Distal thickening of the tibia. Deviating number of tarsal bones: 7 tarsals. Incorrect digit anatomy including missing digits, phalangeal elements, and deformed metacarpal. (o) Misshaped tibia and fibula. Fibula is not completely ossified. Deviating number of tarsal bones: 10 tarsals. Incorrect digit anatomy including missing digits and phalangeal elements. (p) Thickened tibia and fibula. Deviating number of tarsal bones: 8 tarsals. Incorrect digit anatomy including missing digits, phalangeal elements, and deformed metacarpal. Fusion and subsequent re‐splitting of two digits.

Limbs of adult axolotls that have been the subject of serious biting attacks resulting in extensive injuries, tissue tears, and bruising often exhibit abnormal anatomy and even severe deformities once regeneration is complete. The more often limbs are wounded, particularly when regeneration is still ongoing, the more pronounced are the pathological anatomical deviations from the original pattern (Figures 9, 10, 11, 12, 13).

Common malformations are protruding skin flaps, shortened, thickened, and deformed limbs, interdigital skin fusion, as well as missing digits. In severe cases, the limbs are no longer fully functional in their original capacity.

##### Internal examination of limb skeletal anatomy following regeneration

Internal examination of limbs with severe, externally visible pathologies reveals that they frequently also show less serious anatomical skeletal deviations, such as altered numbers of carpal and tarsal bones, abnormal digit anatomy, and bulky long bones in stylopod, zeugopod, and autopod and other tissue structure irregularities. These malformations occur much less frequently during regeneration in the larval stage or after regeneration following controlled amputations with clean cuts.

**FIGURE 14:**
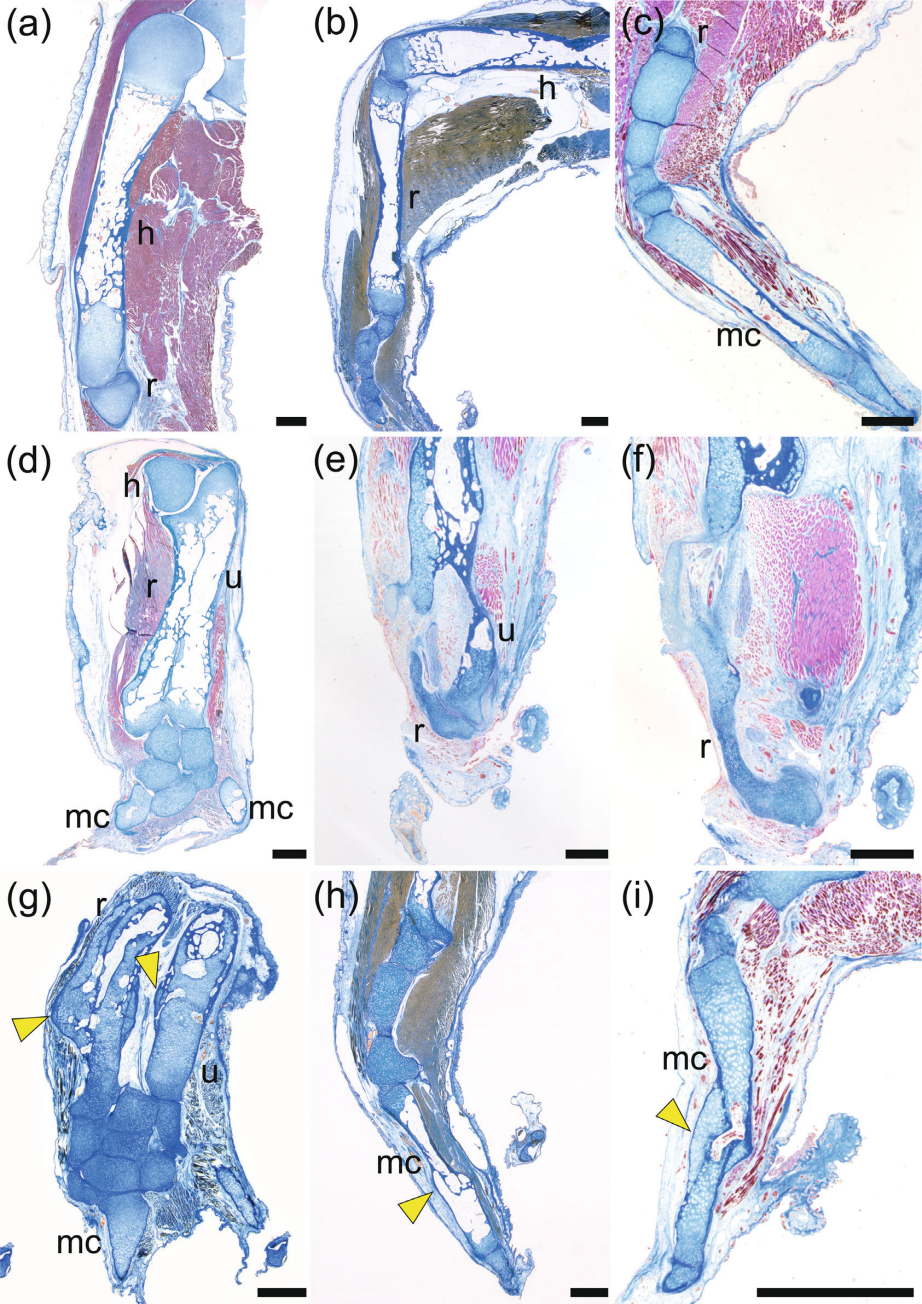
Internal limb skeletal anatomy after regeneration of adult *A. mexicanum*. Histological serial sections of forelimbs stained with Heidenhain's Azan. (a) Unregenerated, normally shaped humerus. (b) Unregenerated, normally shaped humerus and radius. (c) Unregenerated, normally shaped metacarpal. (d) Regenerated zeugopod including fusion of radius and ulna. (e, f) Regenerated zeugopod including malformed long bones. Chaotic tissue structure, no normal differentiation of various tissue types of the anatomical components such as bones, cartilage, and muscles visible. (g) Apparent healing of a fracture in the zeugopods. Bulky cartilaginous callus formation surrounding the original middle parts of the long bones, especially well visible on the radius. (h) Metacarpal, regenerated malformed part surrounding the distal end of the unregenerated normal‐shaped part. (i) Healed fracture of a metacarpal. Cartilaginous callus surrounds and stabilizes the fracture site. Yellow arrows mark the callus outline. h = humerus, mc = metacarpal, r = radius, u = ulna. Scale bars represent 1 mm.

Conspicuous are particularly the numerous fusions of the long bones in the zeugopod. Fusions occur both distally (Figures 9f,h and 13j) and proximally (Figures 9b,d, 10c, 11c, and 13a) and may affect the epiphyses (Figures 9g; 11b, and 13f), or the entire length of the bone (Figures 9a, 13e, and 14d). The absolute length of the long bones in the stylopod and zeugopod could not be determined in micro‐CT scans of unstained limbs (Figures 9 and 10), as only ossified tissue is visualized in those. In axolotl, the diaphysis of the long bones contains a large bone marrow cavity filled with large fatty vacuoles that contain only very few cells that are surrounded by thick calcified cortical bone (cortex). The epiphysis remains cartilaginous (Figure 14a–c) and even at the age of 5–8 years, axolotls still lack secondary ossification centers (Polikarpova et al., 2022), similar to other salamanders (Hanken, 1982; Quilhac et al., 2014). It is therefore not entirely certain whether bones that appear foreshortened (e.g., Figures 9d,e,h,i and 10a, c, f) were actually regenerated with a reduced length or whether the regeneration process did not take place far enough back in time to fully complete the regeneration process or for the epiphyses to have completed ossification. The latter is more likely here, as the animals examined were only about 6 years old. Clearing and staining (Figure 13) as well as 3D models of stained limbs show that regenerated long bones are usually not significantly shorter than unregenerated bones, but within the fused skeletal elements, there is often no well‐defined long bone structure visible (Figures 11, 12, and 14D) due to abnormal bone healing. The mineralized cortical bone surrounding the medullary cavity often exhibits an irregular thickness and gaps (e.g., Figures 13a and 14e,f). Consequently, the shape of the bone marrow cavity, filled with large fatty vacuoles with scattered cells in between, is also misshapen and shows a messy tissue structure. In grave cases, severely deformed long bones occur, which are in fact considerably shortened and misshapen (Figure 13c,g). Moreover, in very rare cases, supernumerary skeletal elements occur in the zeugopod (Figure 13h).

Irregular regeneration processes are also evident in the pronounced pathologies in the autopod area of many limbs. This results, for instance, in a significantly reduced number of carpal bones (Figures 11a,e and 13b, c, f, g), the absence of multiple digits (e.g., Figures 9b, d–f, i, 11a, b, 12c, 13b, c, e–g, i, n, p) as well as branching (Figure 13i,p) and fusion of metacarpal or phalangeal elements, either through connation (Figure 13h) or anatomically abnormal contact points (Figure 13e,p). For detailed information on specific malformations in regenerated limbs, see Figures 9, 10, 11, 12, 13, 14.

In addition to regeneration processes of new more or less functional missing limb parts, internal morphological investigations also reveal the healing of bone fractures caused by bite attacks, in which the limbs were not severed, but long bones were broken instead. Such fractures were healed by the formation of a cartilaginous callus, which connects and stabilizes both bone fragments (Figure 14g (same limb as Figures 9b) and 14i, yellow arrows).

In internal morphological analyses using stained CT scans or histological serial sections, the border between the pre‐amputation old bone and the new distal regenerated area is occasionally clearly discernible (Figures 11d5 and 14h; yellow arrows).

## 4 DISCUSSION

### 4.1 Necessity of non‐model organisms in regeneration research

The primary aim of this investigation was to elucidate the fundamental commonalities and differences of regenerative response in the axolotl and its sister taxon, the tiger salamander. The axolotl has been extensively utilized in regeneration research due to the availability of its genome (Nowoshilow et al., 2018), ease of husbandry and breeding, and the abundance of available biological data. This model organism has provided significant insights into the mechanisms of regeneration, making it indispensable in this field. While the axolotl will continue to play a fundamental role in regeneration research, the inclusion of non‐model organisms is increasingly recognized as being essential for gaining a more comprehensive understanding of regeneration.

The salamander clade encompasses more than 800 species and is particularly valuable for regeneration research. This diversity of non‐model organisms presents great genetic, phenotypic, and ecological variation that is crucial for gaining a broader picture of regenerative abilities, their evolutionary origins, and the role of life history patterns, habitats, or biomechanical demands. Embracing this diversity not only enriches our scientific understanding of this complex process but also holds promise for novel discoveries that can advance both evolutionary and medical research foci.

As a metamorphosing sister taxon of the axolotl, the tiger salamander and the comparison of its regenerative abilities with those of axolotls is particularly interesting. Our results show that tiger salamanders have great regenerative abilities, comparable to those of axolotls, but also display some differences, such as the incomplete healing of bone fractures after injuries. By identifying and characterizing the variation between these species, we hope to expand and advance the understanding of regeneration and provide a framework for future research on underlying mechanisms and genetic variability in different salamander taxa.

### 4.2 Laboratory amputations vs. naturally induced healing and regeneration

In the laboratory, salamanders are typically anesthetized, and limbs are surgically amputated, producing a clean cut that facilitates controlled studies of regeneration. Secondary procedures, such as bone trimming, improve the reproducibility of investigations and ensure that limbs regenerate accurately in experiments (Knapp et al., 2013). Grafting limb tissues can result in abnormalities, such as missing or extra digits (Dunis & Namenwirth, 1977). These abnormalities are also observed in natural populations of different salamander taxa and were for a long time attributed to factors like toxicants, climate change, UVB radiation, and pathogens (Reeves et al., 2013). While these factors primarily disrupt limb development, there is increasing evidence that bite injuries significantly affect limb regeneration in both larvae and adult salamanders (Ballengee & Sessions, 2009; Bothe, Mahlow, & Frobisch, 2021; Bowerman et al., 2010; Sessions & Ruth, 1990; Thompson et al., 2014).

The adult tiger salamanders and axolotls examined in this study, which were kept in groups and repeatedly subjected to conspecific biting attacks despite sufficient space and food, exhibited numerous pathological anomalies in the limb skeletal structure, some of which were exceptionally severe. In a few cases, these serious anatomical changes resulted in restricted or absent limb functionality. A frequently observed pathology in the forelimbs of regenerated axolotl limbs after biting attacks is the fusion of the long bones in the zeugopod (radius and ulna). Forelimbs are often targeted more in bite attacks than hind limbs, possibly because during competitive feeding, the heads and forelimbs of multiple individuals are positioned towards each other, making them prone to accidental or intentional bites. This leads to frequent consecutive injuries, even before previous injuries have healed or severed limb parts have fully regenerated. Such multiple and concurrent injuries cause misaligned and overlapping bone fragments, higher mobility of the bone fragments, arbitrarily bridged connecting parts of the bone fragments, and squeezed and strained soft tissue. This severe disruption of tissue in the limbs leads to strongly affected and disorganized positional information in the cells, yet the positional information of the cells is essential for the exact replacement of missing limb sections and intact regeneration (McCusker & Gardiner, 2013). In injured limbs with bone fractures, the disruption of tissue results in abnormal bone healing involving the formation of a large callus to compensate for the bone fragments' misalignment (Polikarpova et al., 2022), while in severed limbs, this leads to incorrect regeneration processes and severe malformations.

In the larval stage, tiger salamanders show an enormous level of regenerative abilities after bite injuries, which only in rare cases lead to anatomically incorrect regenerates and in these few cases show only slight anomalies. Even after severe injuries, resulting in frayed tissue or protruding bones, larval tiger salamanders exhibit robust regenerative capabilities. Notably, even severely injured limbs that remain attached to the body can lead to full regeneration. Following severe damage, where muscles are severed, nerves are cut, and blood supply is interrupted, the damaged tissue of that limb dies and either completely or gradually sheds off, leaving a flat stump from which regeneration then proceeds normally to produce a nearly perfect replica of the original limb. Contrary to the larvae, the likelihood of serious pathologies in the regenerated limbs increases in subadult and adult individuals. As described above, these anomalies cover a full range from missing and additional skeletal elements to the fusion of long bones in the zeugopod (radius and ulna) to severely deformed skeletal structures in the autopod.

These findings highlight the importance of studying axolotl as well as non‐model organisms in their natural habitats or under husbandry conditions that mimic the natural habitat to gain a comprehensive understanding of the demands on the organism when regenerating after natural bite attacks. This study shows that the regenerative processes and the resulting abnormalities differ between controlled amputations and natural bite attacks and vary along the ontogenetic trajectory.

To understand the impact of bite injuries on limb regeneration, it is essential to determine the likelihood of normal regeneration after such injuries. Consideration should also be given to the fact that under comparatively dense population conditions, whether under natural conditions or in captivity, salamanders often suffer bite injuries in several body appendages at the same time, and thus parallel regeneration processes take place in one animal. The impact of parallel regeneration processes of body parts within a single individual on the speed and accuracy of regeneration remains currently mainly unknown and will require further extensive investigation. Initial investigations revealed that amputation in axolotls triggers systemic activation of progenitor cells, occurring not only in the injured limb but also in contralateral limbs (Johnson et al., 2018). This global response suggests a conserved injury mechanism that, in regeneration‐competent species, is sustained to drive limb regrowth.

A study by Payzin‐Dogru et al. (2023) reveals a previously unrecognized role for body‐wide activation of cell types involved in organismal homeostasis, repurposed for pro‐regenerative responses during vertebrate limb regeneration and provides molecular evidence that limb regeneration relies on the activity of these homeostatic cells. The research also suggests significant implications for understanding how mammals have evolved or retained molecular controls that limit natural regenerative ability. Additionally, the study identifies adrenaline as a key factor in stimulating a systemic stem cell activation response to amputation, involving the peripheral nervous system and adrenergic signaling both at the injury site and in distant tissues. This challenges the traditional view that focuses solely on the injury site, proposing instead that body‐wide stem cell activation is a crucial initial step for regeneration. The data presented here show that contemporaneous regeneration of appendages occurs frequently under natural conditions and may suggest selection for a body‐wide stem cell activation in salamander regeneration.

### 4.3 Metamorphosis as a regeneration‐restricting developmental step

The data clearly demonstrates that tiger salamanders possess remarkable regenerative potential, comparable to that of axolotls, but our investigations have also shown that the quality of limb regeneration decreases with age and especially after completion of metamorphosis in tiger salamanders. In the larval stage, regenerated limbs are virtually indistinguishable from original limbs, both externally and in their internal skeletal anatomy, only very rarely showing anomalies. However, after metamorphosis, regenerative capacity declines sharply. Limb regeneration takes significantly longer, and incidences of visible pathologies, such as supernumerary and extra digits, increase markedly. Although subadult animals are only a few months older than the larvae and are still very young in relation to their average life expectancy, regeneration is no longer as perfect as before metamorphosis.

Much research has focused on understanding why urodeles can regenerate, whereas other vertebrates, such as amniotes, have limited or lost regenerative capacity as adults. Research on appendage regeneration across various tetrapod models has shown a correlation between ontogenetic development and the gradual loss of regenerative ability.

Embryonic and larval anamniotes (fishes and amphibians) possess an almost unlimited capacity for appendage regeneration (Bothe, Mahlow, & Frobisch, 2021; Hutchison et al., 2007; McCusker & Gardiner, 2011), while frogs lose this ability prior to metamorphosis (Dent, 1962). Therein, the data from anurans provide a clear picture that progressive loss of regenerative ability in the limb is a consequence of morphological, cellular, and genomic changes that occur during metamorphosis (Forsyth, 1946; Korneluk & Liversage, 1984; Muneoka et al., 1986; Wolfe et al., 2000; Yakushiji et al., 2007; Yokoyama et al., 2000). In contrast, mammals such as mice and humans can regenerate digit tips (Han et al., 2003; Han et al., 2008), even in the adult stage (Borgens, 1982; Han et al., 2003; Han et al., 2008; Kisch et al., 2015). Overall, the exact mechanisms by which these ontogenetic changes influence the regulation of regeneration are still not well understood.

In line with this, a hypothesis was put forward suggesting that some salamanders, like the axolotl, are paedomorphic—they reach sexual maturity while retaining juvenile characteristics (Tompkins, 1978)—and thus maintain their regenerative abilities, because they do not undergo complete metamorphosis, preserving embryonic‐like cellular characteristics (Galliot & Ghila, 2010). Conversely, the ability to regenerate is maintained in adult fishes (Akimenko & Ekker, 1995; Bothe, Schneider, & Fröbisch, 2021; Darnet et al., 2019; Poss et al., 2003), lizards, although a regenerated lizard tail is missing segmented vertebrae, a fully functional spinal cord, and an organized muscle structure (Alibardi, 2014, 2021; Lozito & Tuan, 2016), and urodeles that complete metamorphosis endogenously (Iten & Bryant, 1973; Wallace, 1981; Young et al., 1983a, 1983b). Regenerative capacities were also demonstrated in axolotls that have been induced to complete metamorphosis in response to experimental activation of thyroid hormone signaling, but metamorphosis significantly reduced the regeneration rate and caused malformations (Rosenkilde et al., 1982; Tompkins & Townsend, 1977). This observation is supported by studies on the regenerative capacities throughout the ontogeny of *Pleurodeles waltl*, which likewise demonstrated a clear reduction in regenerative capability after metamorphosis as measured by both an increase in digit loss and reduction, as well as the overall rate of regeneration (Hua et al., 2023; Wallace, 1981). Additionally, metamorphic blastemal cells of axolotls showed slower cell cycle progression and lower proliferation, establishing the axolotl as a key model for studying the impact of metamorphosis on regeneration (Monaghan et al., 2014). However, metamorphosis in axolotls does not occur naturally and has to be induced artificially, often resulting in other health issues in these animals. It is currently unclear if and how differences between artificially induced versus naturally occurring metamorphosis have an impact on regeneration.

In any case, research on the influence of metamorphosis on regeneration in frogs and salamanders shows that the interplay between these two factors is more complicated than a simple correlation with a life history strategy. Generally, metamorphosis is the regeneration‐restricting developmental step in frogs, while in urodeles it only slows the process of regeneration and makes it more prone to malformations. The loss of regenerative capacities in adult frogs could be the result of the evolution of an extreme larval stage, the tadpole, in frogs and an associated very extreme metamorphosis. While generally comparable in terms of hormonal control and certain morphological changes, salamander metamorphosis is less extreme than that of frogs and may have allowed for the evolutionary maintenance of regenerative capacities whether through active selection or just by chance. Though the correlation between the evolution of extreme metamorphosis and regenerative capacities remains currently speculative, the fossil record lends certain support for this scenario as the capacity for limb regeneration has been demonstrated in the stem lineage of modern amphibians (Fröbisch et al., 2014; Fröbisch et al., 2015).

By all means, the results presented here support the hypothesis that regenerative abilities are not independent of metamorphosis and this complex morphological and physiological transformation acts at least as a limiting developmental biological step for the quality and speed of regeneration. The exact evolutionary and developmental connections between these two systems will have to be further explored in future studies and present an exciting and promising research avenue.

## AUTHOR CONTRIBUTIONS

**Vivien Bothe:** Conceptualization; data curation; formal analysis; investigation; methodology; project administration; validation; visualization; writing – original draft; writing – review and editing. **Nadia Fröbisch:** Conceptualization; funding acquisition; investigation; project administration; supervision; validation; writing – original draft; writing – review and editing.

## FUNDING INFORMATION

The project was funded by a DFG Grant (FR 2647/8‐1) to Nadia Fröbisch.
